# FACTORS INFORMING CLINICIAN CHOICE OF TELEHEALTH MODALITY TO SUPPORT DEMENTIA CAREGIVERS

**DOI:** 10.1093/geroni/igac059.021

**Published:** 2022-12-20

**Authors:** Emma Quach, Emily Franzosa, Christine Hartmann, Lauren Moo

**Affiliations:** VA Bedford Health Care System, Bedford, Massachusetts, United States; Icahn School of Medicine at Mount Sinai, New York City, New York, United States; VA Bedford Health Care System, Bedford, Massachusetts, United States; VA Bedford Health Care System, Bedford, Massachusetts, United States

## Abstract

In summer 2020, 68 clinicians in the New England area caring for Veterans with dementia as part of a specialty team were surveyed regarding caregiver support services, with a 46% response rate (n=31). When faced with the need to abruptly discontinue in-person dementia support services, the majority of respondents offered caregivers support via telephone rather than video telehealth. Only 4 of 31 (13%) mentioned offering video visits for the first time to replace face-to-face visits. Clinician choice of modality largely reflected shifts among preestablished communication modalities/patterns and were influenced by clinician perception that older patients and their caregivers would prefer the telephone. Clinicians without experience using video telehealth with older adults were unlikely to offer video visits despite evidence that many older adults are willing and able to participate. Assessing caregiver wish/ability to participate in video visits may inform and shape clinician choice of telehealth modality.

